# Adjustment of Hydrophobic Properties of Cellulose Materials

**DOI:** 10.3390/polym13081241

**Published:** 2021-04-12

**Authors:** Michael Ioelovich

**Affiliations:** Designer Energy, 2, Bergman Str., Rehovot 7670504, Israel; ioelovichm@gmail.com

**Keywords:** cellulose, crystallinity, modification, cellulose esters, structure, hydrophilicity/hydrophobicity parameters, performance properties

## Abstract

In this study, physicochemical and chemical methods of cellulose modification were used to increase the hydrophobicity of this natural semicrystalline biopolymer. It has been shown that acid hydrolysis of the initial cellulose increases its crystallinity, which improves hydrophobicity, but only to a small extent. A more significant hydrophobization effect was observed after chemical modification by esterification, when polar hydroxyl groups of cellulose were replaced by non-polar substituents. The esterification process was accompanied by the disruption of the crystalline structure of cellulose and its transformation into the mesomorphous structure of cellulose esters. It was found that the replacement of cellulose hydroxyls with ester groups leads to a significant increase in the hydrophobicity of the resulting polymer. Moreover, the increase of the number of non-polar groups in the ester substituent contributes to rise in hydrophobicity of cellulose derivative. Depending on the type of ester group, the hydrophobicity increased in the following order: acetate < propionate < butyrate. Therefore, tributyrate cellulose (TBC) demonstrated the most hydrophobicity among all studied samples. In addition, the mixed ester, triacetobutyrate cellulose (TAB), also showed a sufficiently high hydrophobicity. The promising performance properties of hydrophobic cellulose esters, TBC and TAB, were also demonstrated.

## 1. Introduction

Cellulose is defined as a natural polysaccharide thanks to the works of French chemist Anselme Payen, who isolated it from plant matters and determined its chemical formula [1]. Cellulose is the most abundant biopolymer and organic matter on Earth. Total resources of cellulose in the nature reach one trillion tons [2]. Natural sources of cellulose are all land plants and many algae [3]; in addition, cellulose is found is shells of certain marine creatures [4], and this biopolymer is also synthesized by some microorganisms, e.g., *Gluconacetobacter xylinus* [5].

Various wood species containing 45–50% of cellulose are used as main industrial raw material for production of cellulose. To isolate the cellulose, the wood chips are boiled in boilers under pressure in the presence of delignifying chemicals, such as caustic soda, a mixture of sodium hydroxide with sodium sulfide and sulfurous acid or its salts [6]. Oxidative, organosolv, and some other delignification methods are also used, but on a small scale. As a result, lignin, hemicelluloses and other non-cellulosic components are removed, while cellulose is separated, washed, and dried. Global production volume of wood cellulose is huge and reaches 200 million tons per year [7].

Another commercial cellulose source is cotton containing above 90% cellulose. The world’s production of cotton cellulose is lower than that of wood cellulose, cca 25 million tons per year. The long fibers of cotton cellulose are applied in textile industry, whereas short linter and cotton nap are used for the production of pure cellulose, special paper, microcrystalline cellulose, and cellulose derivatives.

Since cellulose is a renewable and inexhaustible natural raw material, it is widely used for the production of various materials and substances, such as, paper, cardboard, fibers, textiles, films, powdered cellulose, microcrystalline and nano-sized cellulose, cellulose derivatives, composites, and many other products.

Structural studies have shown that cellulose is a linear, stereoregular, semicrystalline polysaccharide composed of repeating anhydroglucose units (AHU), which are linked by chemical β-1,4-glycosidic bonds [8,9,10]. Macromolecules of natural cellulose of various origin may contain 2000 to 30,000 AHU. Long chains of cellulose join together in the lateral direction and form elementary nanofibrils and their bundles, called microfibrils, which contain crystallites and non-crystalline domains. Crystallites of cellulose are inaccessible to water, while non-crystalline domains are easily accessible to water [11]. The content of crystallites in natural cellulose (crystallinity) ranges from 50% (cellulose of herbaceous plants) to 80% (tunicate cellulose) and can be changed via physical (e.g., dry grinding) and physicochemical treatments (e.g., acid hydrolysis) [12].

Each AHU in non-crystalline domains of cellulose contains three accessible hydroxyl functional groups, one primary at C6 and two secondary groups at C2 and C3, which impart an increased hydrophilicity to cellulose materials [10,11,12]. This limits the use of cellulose in such applications as the creation of waterproof and vaporproof materials, the production of hydrophobic fillers and reinforcements compatible with hydrophobic polymers, hydrophobic coatings, paints, adhesives and other hydrophobic materials. Various methods of cellulose modification were used to impart hydrophobic properties including impregnation with solutions of hydrophobic substances, surface hydrophobization, chemical modification, etc. [13,14].

This article discusses several methods for reducing the hydrophilicity and increase the hydrophobicity of natural cellulosic materials, including physicochemical modification that increases the crystallinity of cellulose, and chemical modification by introducing hydrophobic groups instead of hydrophilic hydroxyl groups.

## 2. Materials and Methods

### 2.1. Cellulose 

Pure cotton cellulose of chemical grade (98% α–cellulose, DP = 2700) produced by Hercules Inc., Wilmington, DE, USA, was used as the original cellulose material (OCM).

### 2.2. Acid Hydrolysis

The hydrolysis of OCM was carried out with 1, 2, and 3 M sulfuric acid at 100 °C for 1 h using acid to cellulose ratio 10. After hydrolysis the samples were washed, neutralized with 1% NaHCO_3_, washed again to pH = 7 and dried at 110 °C to constant weight. The resulting samples, hydrolyzed with 1, 2, and 3 M acid, were labelled as HC-1, HC-2, and HC-3, respectively.

### 2.3. Chemical Modification

In order to obtain cellulose derivatives with a high degree of substitution, it is proposed to carry out chemical modification of dissolved biopolymer [14]. Therefore, in this work, the esterification of the original cellulose was carried out in the solutions using trifluoroacetic acid (TFAA) as a solvent and also catalyst. Solutions of cotton cellulose in TFAA were treated with a mixture of TFAA and acetic, propionic or butyric anhydride at 50 °C for 1 h using liquid to cellulose ratio 50. For the preparation of cellulose diesters, diacetate (DAC), dipropionate (DPC), and dibutyrate (DBC), having a degree of substitution (SD) of 1.8–2.2, the anhydride content was 3 M per mole of AGU. To synthesize the cellulose triesters, triacetate (TAC), tripropionate (TPC), and tributyrate (TBC) with SD of 2.7–3.0, the anhydride content was increased to 5 M per mole of AGU. The mixed esters, di- (DAB) and tri–(TAB) acetobutyrate cellulose, was synthesized using mixtures of butyric and acetic anhydrides in molar ratio 2:1. The esters of cellulose were precipitated from solutions with water, and then thoroughly washed, neutralized with 1% NaHCO_3_, washed again to neutral pH-value, additionally rinsed with 50% ethanol and dried at 60 °C in a vacuum chamber to constant weight.

The substitution degree of cellulose esters was controlled by an improved method of chemical analysis [15].

### 2.4. Films of Cellulose Esters

Films of cellulose esters were prepared using 10% solutions of the esters in ethyl acetate. For this purpose, the solutions of cellulose esters were casted into Teflon-covered Petri dishes and placed at ambient temperature to evaporate the solvent and form films. Then, the prepared films were additionally dried at 60 °C in a vacuum chamber to constant weight. 

### 2.5. Coating of Paperboard

Sheets of white paperboard (weight 200 g/m^2^) were coated with 10–20% solutions of cellulose esters in ethyl acetate using laboratory bar coater of Lab Tech Instruments. After coating, the coated sheets were dried at 60 °C in a vacuum chamber to constant weight.

### 2.6. X-ray Diffraction

Samples for X-ray studies were prepared in the form of compressed tablets. The supramolecular structure of the samples was studied using a “Rigaku-Ultima Plus” diffractometer (CuKα–radiation, *λ* = 0.15418 nm). X-ray diffractograms were recorded in the *φ* = 2*Θ* angle range from 5° to 80°. After recording of the diffractograms, the background was separated, and selected X-ray patterns were corrected and normalized. Then diffraction intensities from crystalline (Cr) and non-crystalline domains (NCD) were separated by a computerized method using subtraction procedure of X-ray scattering from NCD (SSNCD) [16,17]. The crystallinity (X, %) of samples was calculated according to Equation [18]

(1) $$X\;=\;100\;\int^{\;}\left(J_{c}\;d\varphi \right)/\int^{\;}(J_{o}\;d\varphi )$$

where *J_c_* and *J_o_* are the corrected and normalized intensities of X-ray diffraction from crystalline domains only and whole sample, respectively.

Interplanar spacings (d) of were calculated by the Bragg Equation

d = 0.5 *λ* (*sin Θ*)^−1^ (2)

Approximate sizes (L) of ordered domains were estimated by the Scherrer’s equation

L = *Kλ* [(*cos Θ*)^2^ (*B*^2^ − *b*^2^)]^−0.5^ (3)

where *λ* is wavelength of X-ray radiation; *B* is experimental width of the peak; *b* is instrumental factor; *Θ* is angle of the peak maximum; and *K* is coefficient close to 1.

### 2.7. Wetting Enthalpy

The enthalpy of wetting (*Q*) of dry samples with water was studied by the method of microcalorimetry at 25 °C using a “TAM III” calorimeter [19]. Three specimens of the same sample were tested to obtain an average result.

### 2.8. Sorption of Water Vapor

The sorption of water vapor was studied at 25 °C using a closed desiccator with a saturated solution of sodium chloride, creating a relative humidity (RH) of 75%. The dry samples were kept in a humid atmosphere with RH = 75% until sorption equilibrium was reached. The sorption value (S, %) was calculated by the equation

S = 100 [(*P/P_o_*) − 1)] (4)

where *P* and *P_o_* are the weight of wet and dry sample, respectively.

Three specimens of the same sample were tested to obtain an average result.

### 2.9. Performance Properties of Materials

The mechanical properties of films of cellulose esters and barrier properties of coated paperboard were tested. Mechanical properties were studied using testing machine “Instron Universal Testing Machine 5900” according to ASTM D 638 and D 828. Testing speed was 5 mm/min. The samples were 50 × 10 mm in size. Five specimens of the same sample were tested to obtain an average result.

Liquid water absorption of the coated paperboard surface was tested by means of Cobb test for 1 h according to TAPPI T-441, whereas grease (oil) resistance of samples (3M Kit Test) was determined according to TAPPI T-559. Three specimens of the same sample were tested to obtain an average result.

## 3. Results and Discussion

### 3.1. Structural Studies of Original Cellulose and Hydrolyzed Cellulose Samples

**Figure 1:**
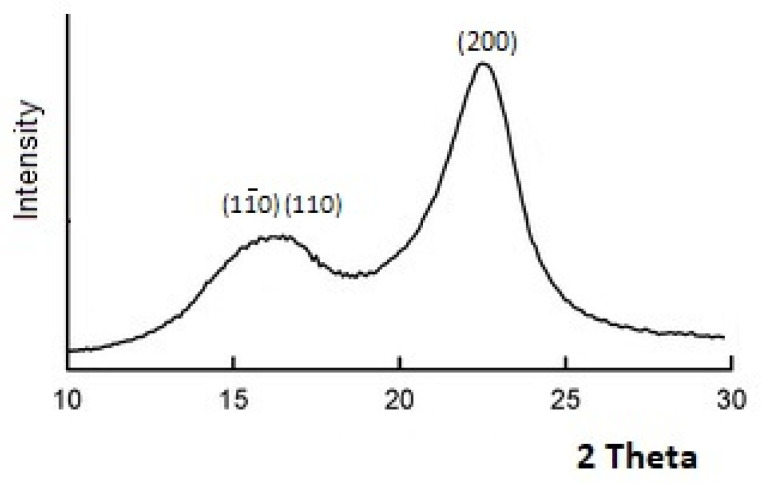
X-ray diffractogram of original cellulose material (OCM).

X-ray study of the original cellulose material (OCM) showed that the diffractogram of this cellulose contains typical crystalline peaks having Miller’s indexes (1–10, (110) and (200) (Figure 1)). 

Calculations according to Equation (1) gave crystallinity value for OCM of about 70% (Table 1). Approximate lateral sizes of crystallites (L) in the direction perpendicular to [200] planes and interplanar spacings (d) between [200] planes of crystalline cell were also calculated.

**Figure 2:**
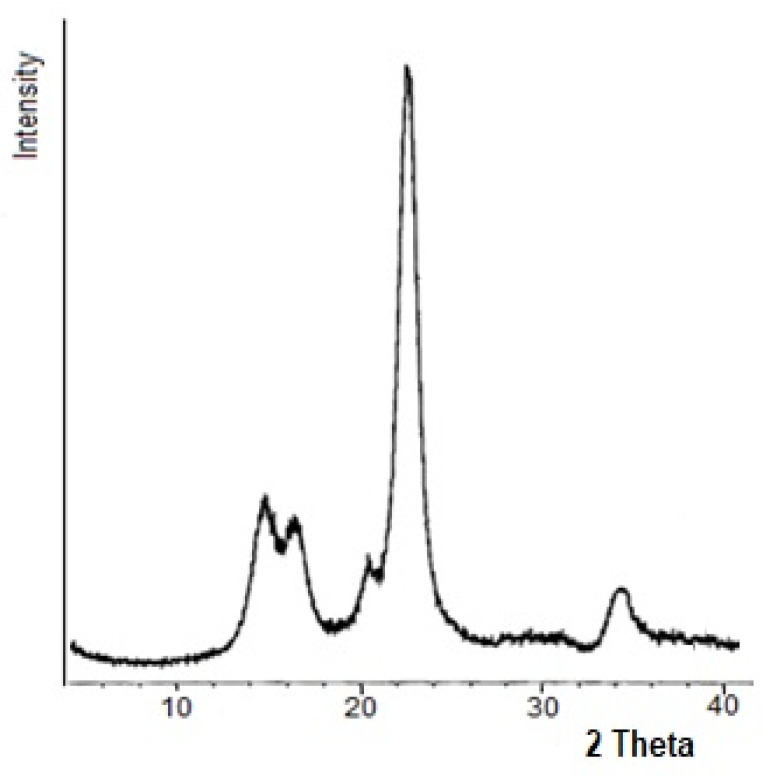
X-ray diffractogram of hydrolyzed cellulose HC-3.

After acid hydrolysis of the original cellulose, the crystallinity increases (Table 1), whereas the crystalline peaks become narrower, and their intensity increases (Figure 2). This is due to the fact that, as a result of acid hydrolysis, a partial removal of non-crystalline cellulose domains and additional crystallization process occur, which lead to increase in the crystallinity and lateral sizes of crystallites [10]. The similar characteristics of crystalline structure using X-ray method with SSNCD were found also for samples of microcrystalline cellulose obtained by acid hydrolysis of cellulose to level-off degree of polymerization [17].

### 3.2. Structural Studies of Cellulose Esters

**Figure 3:**
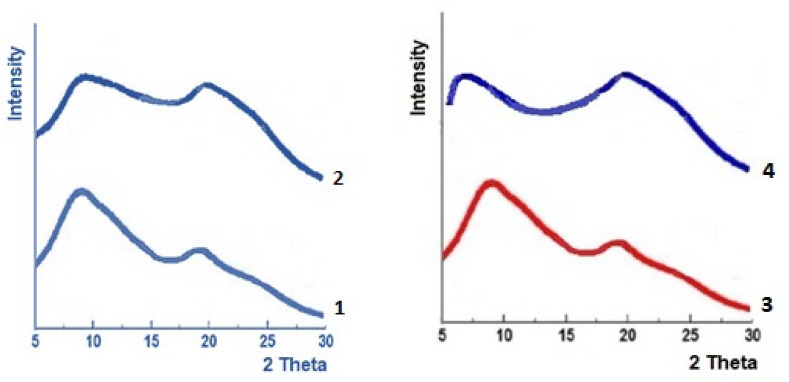
X-ray diffractograms of DAC (**1**), TAC (**2**,**3**) and TBC (**4**).

When replacing the hydroxyl groups of cellulose with ester groups, the supramolecular structure of the obtained cellulose derivatives completely changes. X-ray studies have shown that chemical modification leads to decrystallization of cellulose (Figure 3).

The diffractograms of all obtained cellulose esters show diffuse broad reflexes typical for amorphized polymers having local ordering in the arrangement of repeating units [20]. Such structural organization can be called ‘mesomorphous’ because its order is intermediate between highly ordered crystalline and a completely disordered amorphous structures [21].

For all esters, two X-ray reflexes were observed. The first reflex is located below 10 degrees, and the second reflex is located near 18–20 degrees. Two X-ray reflexes for mesomorphous cellulose esters were found also in papers [22,23]. When the number or type of the ester group changes, the first reflex shifts to the smaller angles, while the angular position of the second reflex remains practically constant.

**Figure 4:**
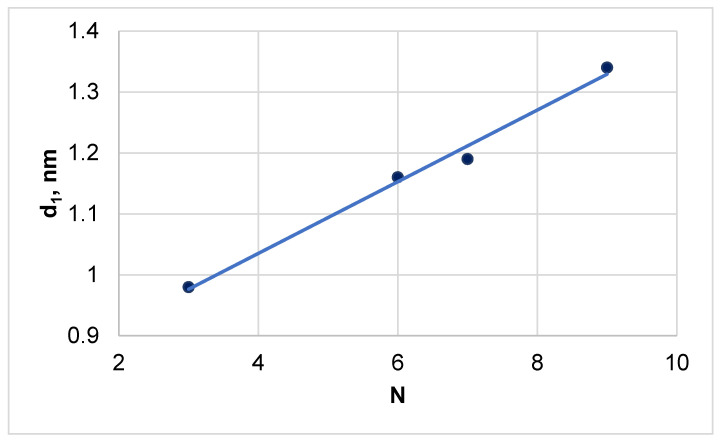
Dependence of interplanar distance (d_1_) on number of non-polar groups (N) in repeating units of various cellulose triesters.

The paper [22] proposed a model of the mesomorphous structure of cellulose esters, according to which the substituted AHUs form layers with an approximately constant distance between them d_2_ = 0.44–0.46 nm corresponding to the angular position of the second reflex. However, in the layer plane, the distance between substituted AHUs (d_1_) depends on the number (N) of non-polar CH_3_ and CH_2_ groups in the repeating units (Figure 4) 

In the present research, for the studied triesters it was found that with an increase of the number of CH_3_- and CH_2_-groups in the substituted AHUs from 3 (TAC) to 9 (TBC), the angular position of the first reflection decreases from 9.0 to 6.6 degrees due to increase in d_1_ from 0.98 to 1.34 nm (Table 2). Thus, the longer the ester substituent, the greater the distance between the links in the layer plane of mesomorphous domains of cellulose triesters (Figure 4).

Calculations also showed that the average sizes of locally ordered domains in cellulose esters are 2–4 nm.

### 3.3. Study of Hydrophilic and Hydrophobic Properties of Cellulose and Its Esters 

The hydrophilic properties of the samples were characterized by their ability to adsorb water vapor (S) and by the thermal effect, enthalpy (Q), after wetting of samples with liquid water. 

The following ratios were used as hydrophilicity parameters

HIP_S_ = *S/S_o_* (5)

HIP_Q_ = *Q/Q_o_* (6)

where *S_o_* and *Q_o_* are sorption value and wetting enthalpy for original cellulose sample.

Using two these characteristics, the average hydrophilicity parameter (HIP) and average hydrophobicity parameter (HBP) were calculated as

HIP = 0.5 (HIP_S_ + HIP_Q_) (7)

HBP = 1 − HIP(8)

The hydrophilic/hydrophobic properties of original and hydrolyzed cellulose samples are presented in Table 3. As it follows from this table, after acid hydrolysis of the original cellulose, the hydrophilicity decreased, and the hydrophobicity increased accordingly.

**Figure 5:**
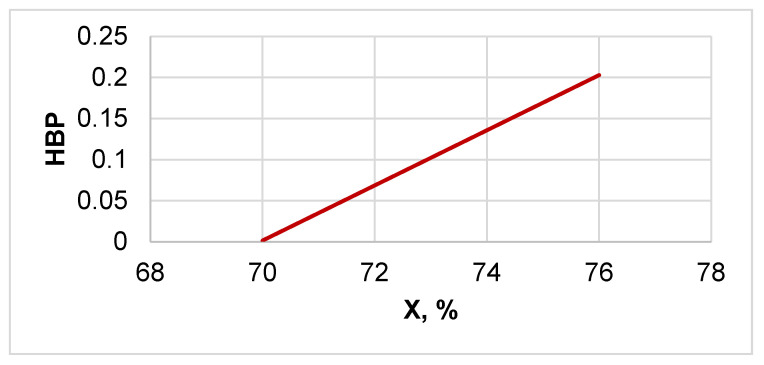
Dependence of hydrophobicity of cellulose samples on their crystallinity.

Furthermore, a direct proportional relationship was observed between the cellulose crystallinity (X) and the hydrophobicity parameter (HBP) (Figure 5). Despite the possibility of increasing the hydrophobicity of cellulose by acid hydrolysis, this modification method turned out to be insufficiently effective, since the increase in hydrophobicity was only 20%.

The study of the chemical modification of cellulose showed that the replacement of hydroxyls with ester groups significantly reduces the hydrophilicity and, therefore, increases the hydrophobicity of the resulting polymer (Table 4), despite the structural amorphization after esterification (Figure 3).

Depending on the type of ester group, the hydrophobicity increases in the following order: Acetate < Propionate < Butyrate. In addition, for the same type of ester group, when passing from diester to triester, a noticeable enhance of hydrophobicity is observed, since in this event all three hydroxyls in the repeating unit are replaced by non-polar groups.

**Figure 6:**
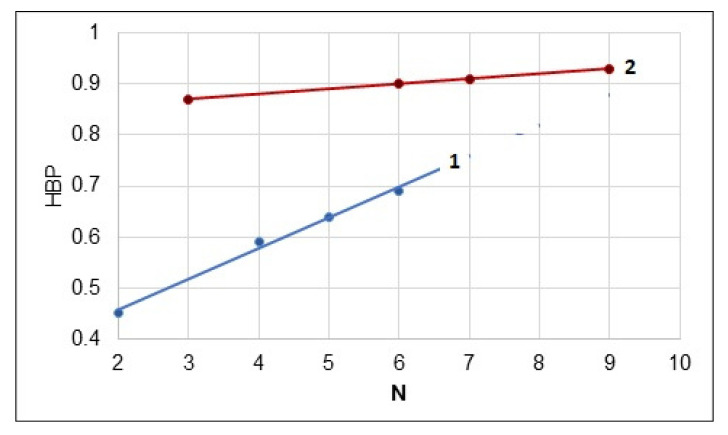
Dependence of hydrophobicity parameter on total number of non-polar groups in repeating unit of cellulose di–(**1**) and tri–(**2**) esters.

If to calculate the total number (N) of non-polar CH_3_ and CH_2_ groups presenting in one repeating unit of cellulose esters, then the following graph can be drawn (Figure 6). As follows from this graph, an increase in the number of non-polar groups in both di- and triesters contributes to rise of their hydrophobicity. The TBC demonstrated the most hydrophobicity among all studied cellulose derivatives. In addition, the mixed triester, TAB, also showed a sufficiently high hydrophobicity.

### 3.4. Performance Properties of Cellulose Esters

The literature reports on a variety of applications for cellulose esters. The market for these esters is permanently growing. In the United States, the growth of the ester market is estimated at more than 6% per year and will reach $12 billion by 2023 [24]. Due to their performance properties the cellulose esters can be applied in such fields as producing of electronic device housings, spectacle frames, anti-fog goggles, cigarette filters, semi-permeable and separating membranes, optical films, heat and rot resistant fabrics, self-cleaning materials, etc. [13,25].

The most important applications of cellulose esters, especially of cellulose butyrates (BC) and acetobutyrates (AB), are compositions of coatings, paints and inks [25,26]. The BC and AB based coatings have such unique performance properties as excellent heat and moisture resistance, as well as good resistance to UV irradiation. These properties are very important for automotive coatings that need a superior exterior durability, as well as for white or pastel coatings of wood that do not yellow when exposed to sunlight.

This article discusses several examples of performance properties of hydrophobic cellulose esters, such as TBC and TAB. 

#### 3.4.1. Mechanical Properties of Hydrophobic Cellulose Esters

**Figure 7:**
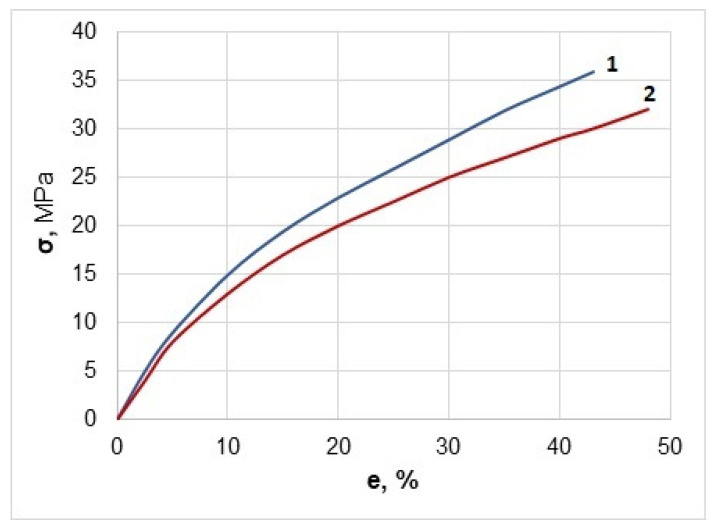
Deformation–stress diagram for TAB (**1**) and TBC (**2**) in dry state.

For this purpose, casted film of cellulose esters were used. The mechanical properties were tested both in dry and wet states after immersion of the samples in water for 24 h. The results showed that although the tensile strength of dry samples was moderate, 32–36 MPa, they had a high elasticity and therefore the elongation at break reached 43–48% (Figure 7).

**Figure 8:**
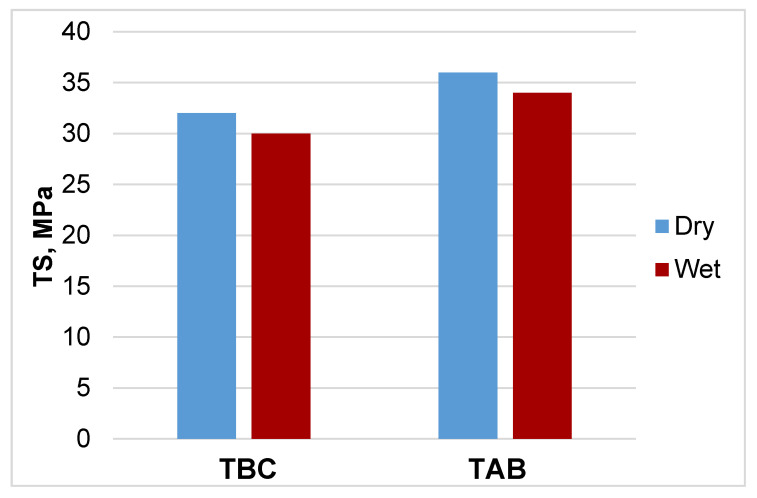
Comparison of tensile strength of samples in dry and wet states.

After exposure to water, the strength of the samples decreased, but this decrease was relatively small and did not exceed 10% due to high hydrophobicity of the studied cellulose esters (Figure 8).

#### 3.4.2. Performance Properties of Coating Layers

Coating the surface of cellulosic materials with various non-polar compositions is considered one of the most widespread and cheap methods of hydrophobization, which has a great application potential [13]. Therefore, here the barrier properties of TBC and TAB coatings on the paperboard surface were investigated.

As can be seen from the obtained results, the original paperboard has no barrier properties, and therefore it is almost completely impregnated with water and oils (Table 5). However, after coating the surface of the paperboard with a thin layer of the cellulose esters, this substrate becomes practically completely protected from the penetration of liquids, water, and grease or oil. 

The grease and oil resistance properties of the cellulose esters are even better than polyolefins, PE and PP, which are permeable to these hydrophobic liquids. In addition, any paper-based substrate laminated with polyolefins cannot be recycled together with paper/board or with plastics. Therefore, such laminates are either landfilled or burned with the garbage. As a result, valuable raw materials are lost.

Unlike polyolefin, laminate layer made of cellulose esters can be easily removed from the surface of paper material using common organic solvents (e.g., acetone, its mixture with alcohol, etc.). After that, the waste paper can be recycled, and the ester solution can be returned to production line and used again for the manufacture of coating compositions.

## 4. Conclusions

Physicochemical and chemical methods of cellulose modification were studied to increase the hydrophobicity of this natural semicrystalline biopolymer. It has been shown that acid hydrolysis of the initial cellulose increases both its crystallinity and hydrophobicity. Furthermore, a direct proportional relationship was observed between the cellulose crystallinity and the hydrophobicity parameter. Despite the possibility of increasing the hydrophobicity of cellulose by acid hydrolysis, this modification method was shown to be insufficiently effective, since the increase in hydrophobicity parameter was only 20%.

A more significant hydrophobization effect was observed after chemical modification by esterification, when polar hydroxyl groups of cellulose were replaced by non-polar substituents. The esterification process was accompanied by the disruption of the crystalline structure of cellulose and its transformation into the mesomorphous structure of cellulose ethers. It was found that the replacement of cellulose hydroxyls with ester groups leads to a significant increase in the hydrophobicity of the resulting polymer. Moreover, the increase of the number of non-polar groups in the ester substituent contributes to the rise in hydrophobicity of cellulose derivative. Depending on the type of ester group, the hydrophobicity increased in the following order: acetate < propionate < butyrate. Therefore, tributyrate cellulose (TBC) demonstrated the most hydrophobicity among all studied samples. In addition, the mixed ester, triacetobutyrate cellulose (TAB), also showed a sufficiently high hydrophobicity.

The promising performance properties of hydrophobic cellulose esters, TBC, and TAB, were also demonstrated. The results showed that dry films made of these esters had moderate tensile strength, but high elasticity. After exposure to water, the strength of the samples decreased, but this decrease was relatively small and did not exceed 10% due to high hydrophobicity of the studied cellulose esters.

The study of the barrier properties showed that because of its high hydrophilicity and porosity, the original paperboard is almost completely impregnated when exposed to both polar (water) and non-polar (grease, oil) liquids. However, after coating the surface of the paperboard with a thin layer of the cellulose esters, TBC or TAB, this substrate becomes practically completely protected from the penetration of polar and non-polar liquids. After usage, the laminate layer made of cellulose esters can be easily removed from the surface of paper material using common organic solvents. Then, the waste paper can be recycled, and the ester solution can be returned to production line and used again for the manufacture of coating compositions.

## Figures and Tables

**Table 1:** Characteristics of crystalline structure of cellulose samples.

Sample	* Yield, %	X %	L, nm	d, nm
OCM	0	70	6.2	0.393
HC-1	96	72	7.0	0.391
HC-2	94	74	8.5	0.390
HC-3	90	77	8.7	0.389

* Note: Mass yield of hydrolyzed cellulose.

**Table 2:** Parameters of mesomorphous structure of cellulose esters.

Sample	N	d_1_, nm	d_2_, nm
TAC	3	0.98	0.45
TPC	6	1.16	0.46
TAB	7	1.19	0.45
TBC	9	1.34	0.45

**Table 3:** Hydrophilic and hydrophobic properties of cellulose samples.

Sample	X, %	S, %	Q, J/g	HIP	HBP
OCM	70	8.6	50	1	0
HC-1	72	8.0	47	0.93	0.07
HC-2	74	7.4	43	0.86	0.14
HC-3	77	6.8	40	0.80	0.20

**Table 4:** Hydrophilic and hydrophobic properties of cellulose esters.

Sample	N	S, %	Q, J/g	HIP	HBP
DAC	2	4.8	27	0.55	0.45
DPC	4	3.5	21	0.41	0.59
DAB	5	3.1	18	0.36	0.64
DBC	6	2.8	15	0.31	0.69
TAC	3	1.2	6	0.13	0.87
TPC	6	0.9	5	0.10	0.90
TAB	7	0.8	4	0.09	0.91
TBC	9	0.5	4	0.07	0.93

**Table 5:** Barrier propertied of coating layers.

Coating Layer	* Coating, %	Water Absorption, Cobb Test, g/m^2^	Grease (Oil) Resistance, Kit Test No. (%)
No	0	98	1 (0%)
TBC	5	0.4	11 (95%)
	10	0.1	12 (100%)
TAB	5	0.6	11 (95%)
	10	0.2	12 (100%)

* Note: Coating percentage = 100% × (weight of coating/weight of paperboard).

## Data Availability

Not applicable.
